# Retraction Note: Long non-coding RNA AGAP2-AS1, functioning as a competitive endogenous RNA, upregulates ANXA11 expression by sponging miR-16-5p and promotes proliferation and metastasis in hepatocellular carcinoma

**DOI:** 10.1186/s13046-022-02521-z

**Published:** 2022-11-02

**Authors:** Zhikui Liu, Yufeng Wang, Liang Wang, Bowen Yao, Liankang Sun, Runkun Liu, Tianxiang Chen, Yongshen Niu, Kangsheng Tu, Qingguang Liu

**Affiliations:** Department of Hepatobiliary Surgery, The First Affiliated Hospital of Xi’an, Jiaotong University, 277 Yanta West Road, Xi’an, 710061 China

**Retraction Note: J Exp Clin Cancer Res 38, 194 (2019)**

**https://doi.org/10.1186/s13046-019-1188-x**

The Editor-in-Chief has retracted this article. After publication, concerns were raised regarding high similarity between the sh-Control Invasion image in Fig. 10C and the si-Control Invasion image in Fig. 5E in the authors' other article ([1], now retracted) published within a similar time frame. Further investigation by the Published has identified that the sh-Control Invasion image in Fig. 10C also appears to overlap with the HCCLM2-miR-16-5p EV image in Fig. S4B.

Additionally, the article appears highly similar to another article from an unrelated group ([2], now retracted).

The Editor-in-Chief therefore no longer has confidence in the presented data.

None of the authors have responded to any correspondence from the publisher about this retraction notice.
